# Multiple Scales of Control on the Structure and Spatial Distribution of Woody Vegetation in African Savanna Watersheds

**DOI:** 10.1371/journal.pone.0145192

**Published:** 2015-12-14

**Authors:** Nicholas R. Vaughn, Gregory P. Asner, Izak P. J. Smit, Edward S. Riddel

**Affiliations:** 1 Department of Global Ecology, Carnegie Institution for Science, Stanford, United States of America; 2 Scientific Services, South African National Parks, Skukuza, South Africa; 3 Centre for African Ecology, School of Animal, Plant and Environmental Sciences, University of the Witwatersrand, Private Bag 3, Wits 2050, South Africa; 4 Conservation Management, South African National Parks, Skukuza, South Africa; 5 Centre for Water Resources Research, University of KwaZulu-Natal, Pietermaritzburg, South Africa; Technion—Israel Institute of Technology, ISRAEL

## Abstract

Factors controlling savanna woody vegetation structure vary at multiple spatial and temporal scales, and as a consequence, unraveling their combined effects has proven to be a classic challenge in savanna ecology. We used airborne LiDAR (light detection and ranging) to map three-dimensional woody vegetation structure throughout four savanna watersheds, each contrasting in geologic substrate and climate, in Kruger National Park, South Africa. By comparison of the four watersheds, we found that geologic substrate had a stronger effect than climate in determining watershed-scale differences in vegetation structural properties, including cover, height and crown density. Generalized Linear Models were used to assess the spatial distribution of woody vegetation structural properties, including cover, height and crown density, in relation to mapped hydrologic, topographic and fire history traits. For each substrate and climate combination, models incorporating topography, hydrology and fire history explained up to 30% of the remaining variation in woody canopy structure, but inclusion of a spatial autocovariate term further improved model performance. Both crown density and the cover of shorter woody canopies were determined more by unknown factors likely to be changing on smaller spatial scales, such as soil texture, herbivore abundance or fire behavior, than by our mapped regional-scale changes in topography and hydrology. We also detected patterns in spatial covariance at distances up to 50–450 m, depending on watershed and structural metric. Our results suggest that large-scale environmental factors play a smaller role than is often attributed to them in determining woody vegetation structure in southern African savannas. This highlights the need for more spatially-explicit, wide-area analyses using high resolution remote sensing techniques.

## Introduction

Both grass and woody plant vegetation contribute to the primary production of savannas, yet mixtures of these diverse growth forms persist along a broad spatial and temporal continuum in these ecosystems [1]. Vegetation growth form and structural composition within any given savanna landscape results from a multitude of interacting, multi-scale influences [2,3]. Climate plays a dominant role in determining regional patterns of savanna vegetation. Savannas are often classified as arid or semi-arid [4,5], and the maximum potential tree cover is often linked to annual rainfall [1,6]. However, within any given rainfall regime, tree cover and structure also greatly depend upon the hydrologic features of a site [7].

Because of their physical interaction with water [8], geologic substrate and soils are also major influences on savanna vegetation structure. Parent material affects soil texture and fertility, resulting in regional-scale variability of vegetation structure [9]. Yet on a given geologic substrate, variable rates of weathering over time result in changing edaphic conditions [10,11]. For example, catenas are a common feature in African savanna landscapes, resulting from differential hydrologic conditions along hillslopes [12]. Gradients of vegetation structure are thus often found along catenas [13,14].

Within a given substrate and climatic regime, other factors significantly affect vegetation structure. The relative abundance of grass and woody plants in a savanna system results from the differential response of these two growth forms to the environment and to one another [15]. A warm dry season combined with significant grass biomass can lead to the frequent occurrence of fires, which are widely implicated in the maintenance of savanna physiognomy [16,17]. Fire suppression can alter the grass to woody vegetation ratio [18]. Herbivores can also have large effect on woody vegetation cover [19], and this effect can exceed that of fire [20,21]. Near human settlements, range use and wood collection can significantly alter the structure of a savanna landscape [22,23]. There are yet other factors that play a role in determining savanna vegetation structure, and the effects of these drivers are not independent [24,25]. For example, soils affecting vegetation structure will also affect fire behavior [26], and fires can, in turn, modify soil properties [27] and interact with herbivore patterns [16,28].

Despite numerous studies, these basic patterns of change in savanna vegetation cover are only understood at either broad or very fine spatial scales. We know little about ecological scales at which these factors operate, or how they generate the vegetation patterns observed on a given landscape. To fill this knowledge gap, a large and spatially extensive amount of fine-scale data is needed, as multiple scales must be covered simultaneously. Remote sensing is currently the best way to collect such a dataset. To this end, Bucini et. al. [29] combined optical and radar satellite data to gain insight into vegetation cover response to many of the above factors in the Kruger National Park, South Africa One key finding was that inclusion of spatial coordinates into the model more than doubled the variance explained, indicating that much of the variation occurs at landscape scales than is often considered.

To more fully understand drivers of savanna vegetation structure, it may be advantageous to look more closely at landscape-scale patterns. In this context, the Kruger National Park has designated four watersheds, representing two distinct geologies (granites, basalts) and two common savanna rainfall regimes (425, 550 mm yr^-1^), as science-community research areas for the study of savanna ecological patterns and processes under nested drivers of changing woody vegetation cover and structure [30]. Using airborne Light Detection and Ranging (LiDAR), we mapped the three-dimensional vegetation structure and topography of these four contrasting watersheds to answer two questions: i) At what spatial scales does savanna vegetation structure vary, and what factors are influencing the observed patterns? ii) How do these patterns differ across the four contrasting watersheds? The answers to these questions will improve our understanding how drivers of vegetation structure interact at multiple ecological scales, and how changes in environment may affect the vegetation on a particular landscape.

## Methods

### Study Site

Kruger National Park (KNP), which covers an area of nearly 2 million ha, was first set aside as a game reserve by the government of South Africa in 1898. The park covers the region between 25° 30’ and 22° 19’ S latitude, and between the Great Escarpment to the west and the Mozambique border to the east. The region has hot summers and mild winters, with maximum daily temperatures commonly above 30° C in the summer and minimum temperatures rarely below 5° C during the winter. Rainfall at KNP is highly seasonal, mostly occurring between October and April, with a general gradient of decreasing rainfall ranging from approximately 600 mm yr^-1^ in the south to 400 mm yr^-1^ in the north [31]. With few exceptions, the topography of the park is made up of gradually undulating hillslopes (on granites) or plains (on basalts). While the topography is gentle, the underlying geology of the park is very diverse [32], and it has influenced the formation of a variety of soils across the landscape. The effects of these soil changes are reflected by changes in the vegetation [10,32].

**Fig 1:**
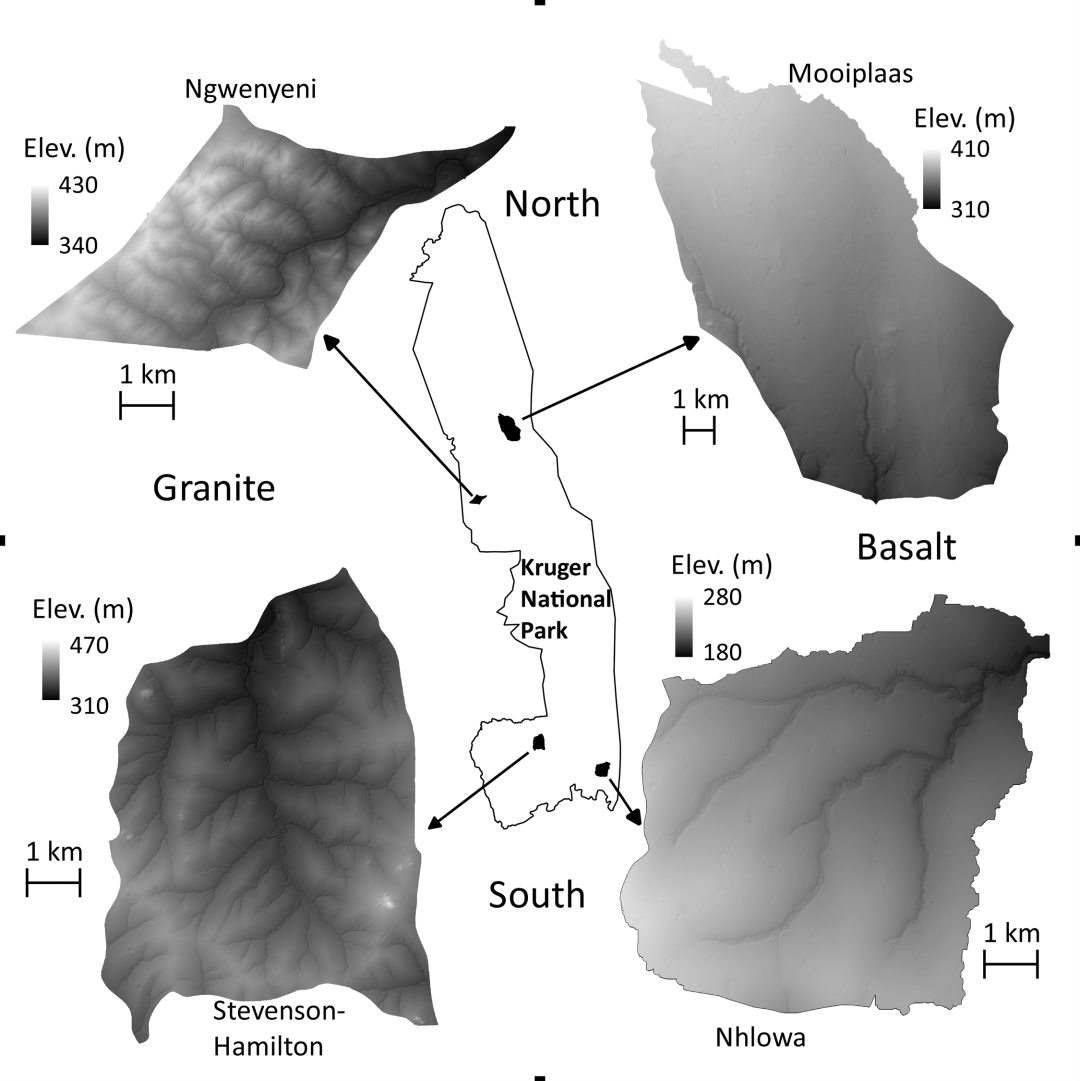
Map of the four focal watersheds. The four study watersheds in Kruger National Park. A LiDAR-derived ground elevation map is shown for each site, demonstrating the hydrologic and geologic differences between the watersheds.

Four watersheds within non-manipulated regions of KNP have recently been designated as long-term research areas intended to further our understanding of the ecological processes operating at landscape scales [30]. Each of these four watersheds represents at least one entire third-order stream watershed (Fig 1). These watersheds represent the two most dominant geologies in the park—basalt and granite formations—as well two contrasting levels of rainfall (S1 Table), spanning a large share of conditions found throughout the park.

### LiDAR Data Collection and Height Models

Between April 22 and May 3 of 2012, each of the four watersheds was surveyed by the Carnegie Airborne Observatory-2 (CAO-2) [33] using a LiDAR system. Survey flights were completed under full permission from South African National Parks (SANParks) and Kruger National Park Air Services. Nominal survey altitude was 2000 m and airspeed was 100 kts (51.4 m s^-1^). The LiDAR was set to a pulse frequency of 100 kHz and a scan frequency of 18 Hz. With 50% overlap and up to four returns per pulse recorded, the resulting datasets had an average point spacing of 0.59 to 0.79 m (inversely, 1.61 to 2.85 points per m^2^). LiDAR point positioning errors were <15 cm vertical and < 26 cm (σ) horizontal.

With the LiDAR point data for each watershed, we developed maps of both ground elevation and vegetation height relative to ground at 1.12 m x 1.12 m resolution (the nominal point spacing of a single flight line of LiDAR data under our settings) using the LAStools software suite (Rapidlasso GmBH; Gilching, Germany). Prior to any further analysis, areas of animal enclosures/exclosures and other experimental plots, as well as large rock outcrops occurring within the watersheds, were removedfrom the vegetation height and ground elevation maps.

### Vegetation Structure

To best represent the diversity of vegetation structure within the watersheds, we used a stratified sampling approach to select vegetation sample points. We divided the cells in the vegetation height map for each watershed into five strata, based upon the observed height model values for each map. Stratum boundaries were defined using the minimum, maximum, and the 20^th^, 40^th^, 60^th^, and 80^th^ percentiles from each watershed. For each watershed, we randomly selected 1000 cells from each stratum, and used the centers of these 5000 total cells as sample points for computing vegetation structure metrics.

We computed six metrics from the LiDAR canopy height models to define vegetation structure. Other components of structure, such as branch and leaf traits, are functionally important but not considered here. Within a 15 x 15 cell (16.8 x 16.8 m) window surrounding each sample location, we counted the number of vegetation height model cells falling into each of four height classes: 0.5 to 2.5 m (shrub), 2.5 to 5.0 m (small tree), 5.0 to 10.0 m (medium tree), and greater than 10.0 m (large tree), because experience suggested that the four classes of woody vegetation would respond differently to changing environmental conditions. By dividing by 225 (the number of cells in the widow), these counts were converted to proportion of window area. This window size was found to be optimal for obtaining a full range of proportion values between 0 and 1 for each height class. Additionally, we used the maximum height value for the same window as an indicator of total vegetation height. Finally, we computed a crown density at each sample point as well. First, a map of individual crown locations was built using a simple local maximum search algorithm [34]. To reduce the detection of multiple crowns from a single tree, we first smoothed the vegetation height model with an 9 x 9 cell Gaussian kernel, where the optimal size of this kernel was determined by visual inspection. We considered all cells of the smoothed map with a greater height than all 8 neighbors as crown tops. Using all crown tops greater than 1.0 m in smoothed height, we computed the crown density within a circular window of 50 m radius surrounding each sample point.

### Environmental Variables

We mapped several environmental variables within the watersheds using the LiDAR-derived elevation models. We used the Terrain Analysis Compound module (ta_compound) of the SAGA GIS software package version 2.1.0 (SAGA User Group Association; Hamburg, Germany) to identify and map slope (SLP), aspect (ASP), and horizontal distance to the nearest first-order stream (DO1), second-order stream (DO2), and third-order stream (DO3). Additionally, we computed two metrics to capture the influence of hillslope catenas. These metrics were Relative Slope Position (RSP), defined as the height of a given location above the nearest downslope channel relative to the height of the nearest upslope crest above the same point, and Topographical Wetness Index (WET) [35]. In essence, WET is a measure of saturation frequency incorporating both local water storage area and the contributing area of stream input. We also had access to yearly fire scar extents mapped across the KNP for all years from 1955 to 2012 [26,36]. Using these data we calculated Average Fire Return Interval (FRI) as 58 divided by the number of fires that occurred, and we calculated Fire Wait Time (FWT) as the number of years from most recent fire. Finally, for one of the watersheds, we included a map of the hydrological soil response within the southern granite watershed [37]. Soil type (STP) was classified into one of seven classes: alluvial, clayey interflow, clayey recharge, rock outcrops, sandy interflow, sandy recharge, and sodic.

### Spatial Scale Analysis

The variation of each vegetation structural metric in these systems could be examined at two spatial scales. The data were studied first at a larger regional-scale level by comparing the four whole watersheds directly. Multiple simple linear models were used to investigate how the structural metrics respond to the regional-scale factors of geology and climate: one model comparing the climate regions, North and South, one model comparing the geologic substrate, and one model with both of the above factors along with a term for their interaction. The R^2^ value for each of these models was computed to compare the relative influence of each factor on vegetation structure. At a second scale of analysis, the data were compared within watersheds to determine trends at distances ranging from a few meters up to several kilometers. At this level, we first computed variograms for each of the metrics within each of the watersheds. These variograms provided a measure of decreasing similarity in vegetation structure between sample locations across increasing distances between locations. Using these variograms we could assess how the different vegetation metrics responded to the same environment and how these responses varied between watersheds.

### Model Design

At the within-watershed level, we sought to determine the proportion of vegetation structural variance driven by known and measureable environmental factors. We modeled each of the six vegetation structural metrics as response variables using a generalized linear model framework. Proportion of shrub, small tree, medium tree and large tree vegetation were modelled with an assumed binomial error distribution with a logit link function. No weighting of individual samples was used, as the number of cells from which this variable is computed remained constant for all sample locations. Maximum height was modelled with a normal distribution and identity link function. Crown density, being count data, was modelled with a Poisson distribution and log link function. The explanatory variables used in each model included the full set of environmental variables as well as all one-way interactions of these variables. The fit statistics for each full model were stored for later comparison.

Early analysis of the residuals in the models suggested that significant spatial covariance remained between the samples for the models for most response-watershed combinations. Therefore, we added an autocovariate (ACV) term to each model to both remove the influence of this variation and assess the total amount of explainable variation within the data that our initial models failed to fit. The autocovariate is a variable computed from the weighted average of the residual values of all neighboring samples that can be added to model the autocorrelation explicitly [38]. In each model, the ACV term was computed using the function autocov_dist in the spdep library [39] of the R programing language [40]. The ACV computation requires one parameter, the maximum distance within which to search for neighbors. To keep this objective between models we used the maximum of 50 m or the distance between samples at which a correlogram showed only 10% of the correlation remained between the full model residuals for each model.

## Results

**Fig 2:**
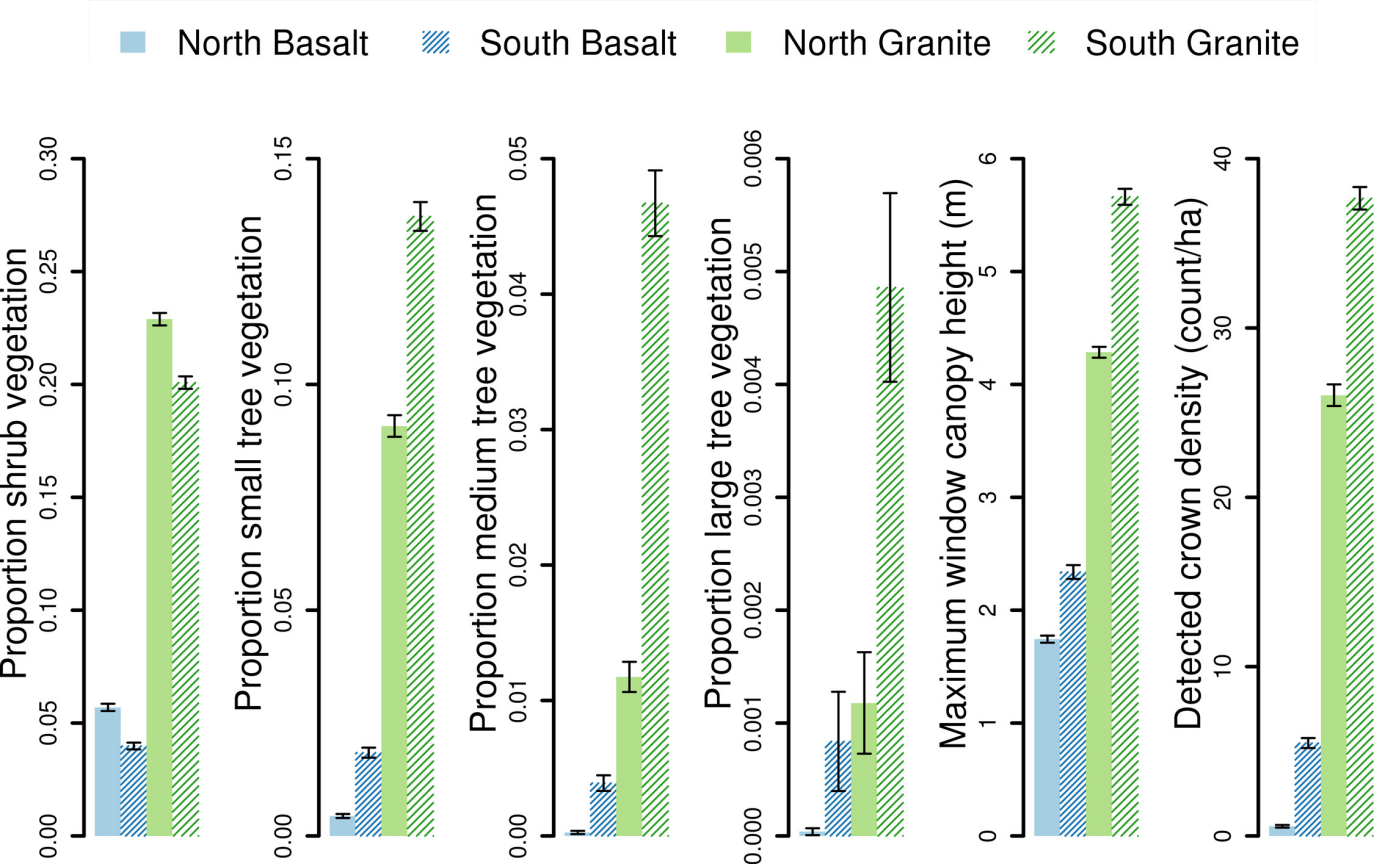
Mean values of the vegetation structure metrics for the four watersheds. The mean and 95% confidence interval for each of the six vegetation structure metrics across 5000 randomly located samples selected from each watershed (without stratification). The vegetation structure within the four watersheds can differ by an order of magnitude. Proportion vegetation is defined as the proportion of area in a 16.8 x 16.8 m window of the vegetation height map that is in the given height class: 0.5 to 2.5 m (shrub), 2.5 to 5.0 m (small tree), 5.0 to 10.0 m (medium tree), and >10.0 m (large tree).

We discovered large differences in woody vegetation between each of the four watersheds. Average values of the vegetation response variables differed by up to an order of magnitude (Fig 2). The two granite watersheds had significantly greater cover and taller woody vegetation compared to the two basalt watersheds. Within geologic substrate, rainfall also had a detectable effect on vegetation structure, especially the amount of taller vegetation present (Fig 2). Medium- and large-sized tree vegetation was nearly non-existent in the dry (northern) basalt watershed, while at the other extreme nearly 5% of the vegetation cover in the wet (southern) granite watershed was made up of these two tree classes. Additionally, total annual rainfall had minimal effect on the total amount of shrub vegetation, with drier landscapes having 2—\3% more cover than wetter landscapes on the same geology. For all trees ≥2.5 m cover was significantly greater in wetter landscapes than in the paired geological sites of lower rainfall. The total amount of vegetation cover of all height classes also showed little response to rainfall, summing to 6.2% and 6.3% for northern basalt and southern basalt and 33.3% and 38.9% for northern granite and southern granite, respectively. However, the relative amounts of short and tall woody vegetation varied strongly by both rainfall and geologic regime.

Modelling differences in vegetation structure between watersheds, we found that the relative strengths of substrate and climate as explanatory factors were dependent upon the metric applied. Overall the total variance explained with both factors in the model ranged from 1% to >50% (Table 1). These broad-scale factors performed best with vegetation metrics describing the cover of shrubs and smaller trees as well as the height and distribution of all woody vegetation. For medium and large trees, however, climate and substrate explained very little of the variance across the watersheds. Even when all terms were included, these models did not exceed an R^2^ of 0.13. In all models, it was the difference in geologic substrate that explained more of the variance amongst the four watersheds, as climate alone could never explain more than 4% of total variance.

**Table 1:** Proportion of variation (R^2^) explained by geology and rainfall in models for each of the six vegetation structure metrics.

Model Factors	P(Shrub)	P(SmTree)	P(MedTree)	P(LgTree)	Max.Height	Density
Rainfall^a^	0.010	0.027	0.035	0.004	0.001	0.033
Geology	0.521	0.315	0.069	0.005	0.338	0.398
R * G^b^	0.531	0.351	0.127	0.010	0.347	0.436

^a^North and South watersheds receive lower and higher rainfall, respectively

^b^This model includes the two main factors and an interaction term

**Fig 3:**
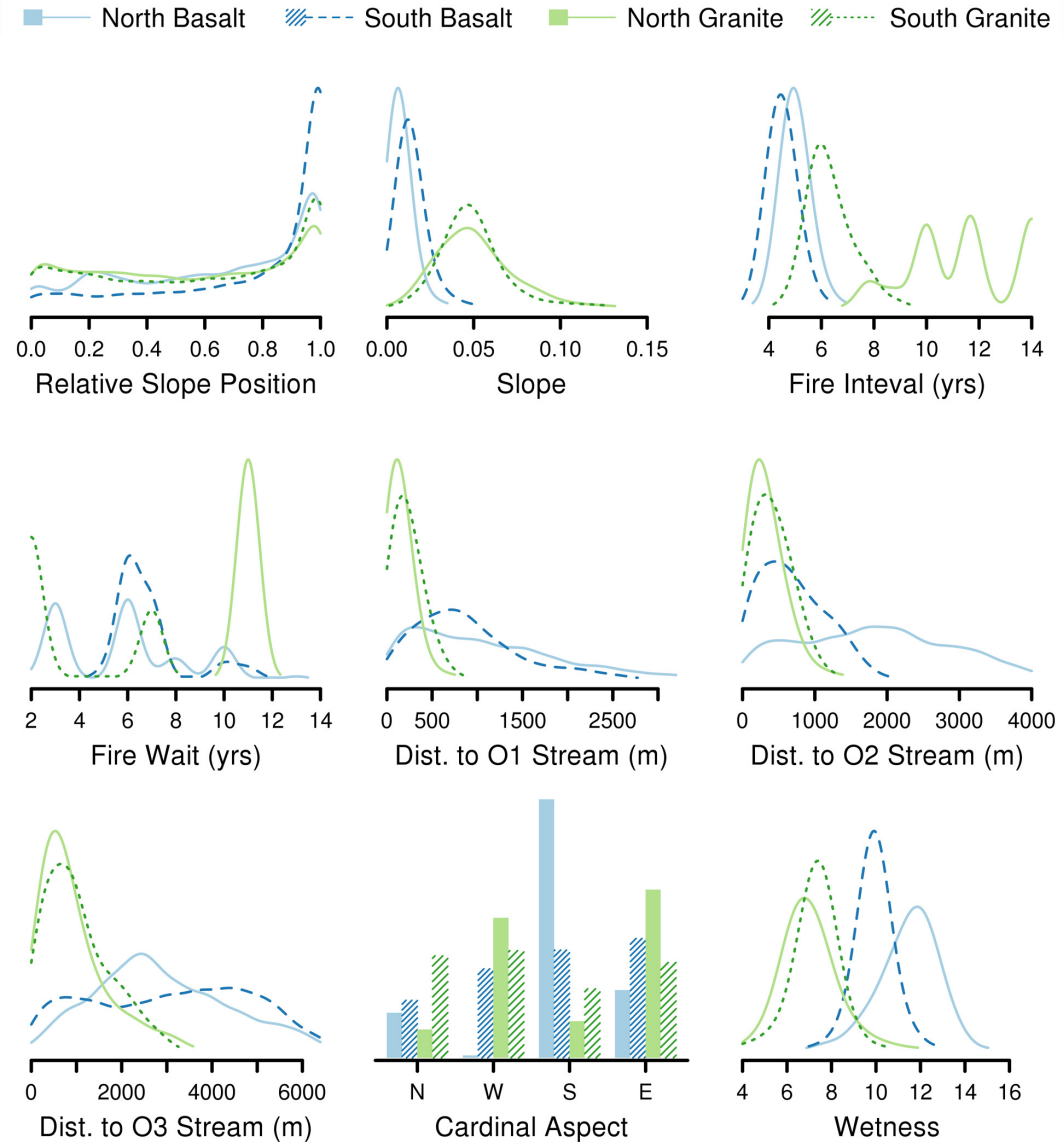
Distributions of the environmental factors by watershed. Probability density functions of environmental drivers of woody vegetation cover showing the large differences between watersheds. The distributions are computed using a Gaussian kernel on 5000 randomly located samples selected from each watershed (without stratification), and they are scaled to fit all four watersheds on a single plot. The abbreviations O1, O2 and O3 refer to order 1, 2 and 3 streams, respectively.

The large observed differences in vegetation structure were paralleled by the differences in environmental variables between watersheds (Fig 3). Topographic differences between the watersheds were much larger between the two geologies than between high and low rainfall. Granite substrate was associated with steeper slopes and more of the total area at valley bottom (RSP near 0) and hillcrest (RSP near 1) than on the face of a slope. The shapes of hillslopes in the basalt watersheds were quite different, with a much greater proportion of the total area at or near hillcrest, as well as much greater distances to all orders of stream. Similarly, in the basalt watersheds, the hillcrest and stream channels are much further apart, often separated by more than 1 km. Topographic wetness index (WET) values were greater, on average, in the basalt watersheds as well, and this was much more so in the drier northern watershed. This reflects both the flatter terrain (greater pooling potential) and wider basins (larger inputs) observed in the basalt watersheds. There were also large differences in the fire regimes of the four watersheds, with fires occurring more frequently in the basalt watershed than in the granite. Likewise, wetter watersheds experienced more frequent fires. The dry granite watershed in particular had very infrequent fires, ranging from 8 to 14 years between fires, as well as the associated longer wait times from the last recorded fire.

**Fig 4:**
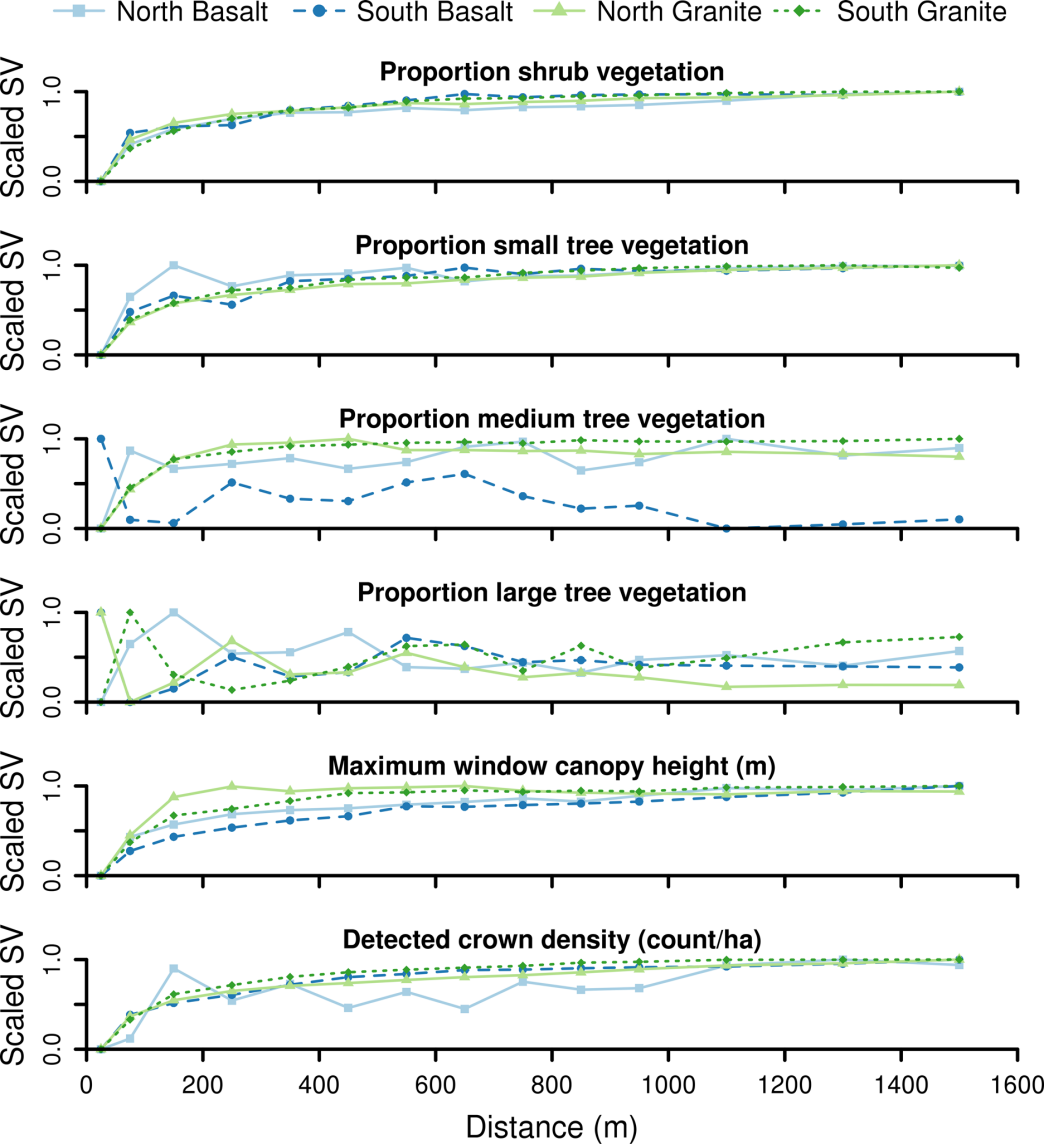
Spatial covariance of the vegetation structure metrics. Variograms showing the extent of spatial covariance of the six vegetation structure metrics for each of the four watersheds. In each of the variograms, semi-variance (SV) is scaled to span the interval [0,1] for better comparison of the range of spatial dependence between watersheds. Proportion vegetation is defined as the proportion of area in a 16.8 x 16.8 m window of the vegetation height map that is in the given height class: 0.5 to 2.5 m (shrub), 2.5 to 5.0 m (small tree), 5.0 to 10.0 m (medium tree), and >10.0 m (large tree).

We found that spatially-driven covariance was sizeable for most model-watershed combinations (Fig 4). The variograms for each vegetation structure variable were generally consistent between watersheds for shorter woody vegetation as well as for density of tree crowns. Maximum covariance was usually reached by a distance between 200 and 1000 m. However, the variograms for maximum canopy height did differ between the watersheds, showing a much shorter range of association (150 m) for the drier granite watershed, and a longer range (1 km) for the basalt watersheds in general. The cover of taller vegetation—medium and large tree on basalts and large tree on granites—seemed to have very little spatial structure at all, as evident from the random-appearing nature of these variograms.

**Fig 5:**
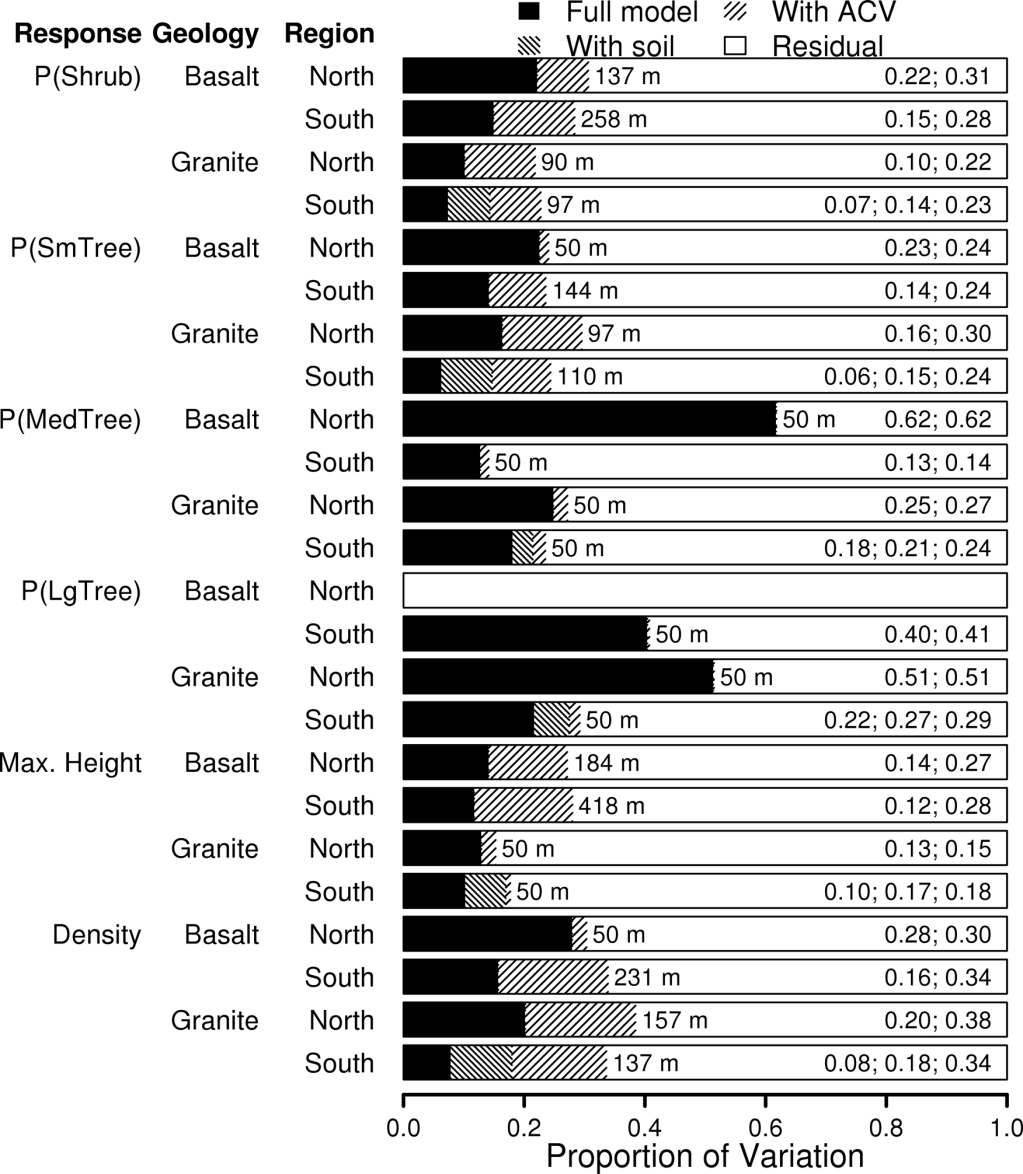
Variance explained by the models for the four watersheds. The proportion of variation, measured as R^2^, for all combinations of response variable and watershed. Values are given both before and after an autocovariate (ACV) was added to a full model with all environmental variables and one-way interactions. For the southern granite watershed, the total proportion of variance explained with soil class (STP) in the model—prior to the addition of and ACV term—is also shown. Bars represent the R^2^ values given on the right side. The lag distance used to compute the ACV term for each model is given after each bar. One row is missing because there was too little large tree vegetation on the northern basalt site to fit a model. P(Shrub), P(SmTree), P(MedTree) and P(LgTree) refer to proportion of area in a 16.8 x 16.8 m window of the vegetation height map that is in the given height class: 0.5 to 2.5 m (shrub), 2.5 to 5.0 m (small tree), 5.0 to 10.0 m (medium tree), and >10.0 m (large tree).

The finer-scale environmental variables combined to explain little of the total variation for most models, with R^2^ values rarely reaching or exceeding 0.3 (Fig 5). Under the full models, and prior to adding the ACV term, the range of spatial autocorrelation of the residuals was in the range 200–500 m for all models (Fig 5), much shorter than the 1 km distance of autocovariance found for raw structure data. After including the ACV term, the autocorrelation had disappeared from the model residuals (S1 Fig). Additionally, the R^2^ values of most models improved substantially with the addition of the ACV term, in some cases more than doubling. In general, the cover of shrub and small tree vegetation, the total canopy height, and the crown density were poorly modeled, and it is for these variables that the ACV term helped the most. The proportional cover of medium and large tree vegetation (both > 5 m) was better explained by the models than the other structural variables. Additionally, the inclusion of an ACV term did little to improve these models. For the southern granite watershed, where soil textural class (STP) information was available, inclusion of this term provided noticeable improvement to the models (Fig 5). The gains in R^2^ from adding this variable alone were 2–5 times larger than for any other single variable, and sometimes added more than all other factors combined (S2 Fig).

## Discussion

Given prior knowledge of the importance of geologic substrate and rainfall to vegetation structure [10], large differences in vegetation height, cover and density were expected between watersheds. While this expectation was met for all vegetation structure metrics, the differences in their magnitudes were notable. Interestingly, substrate had a much larger effect than rainfall on the vegetation cover of shrubs and the smaller tree class across the rainfall range in our study region. In a semi-arid savanna system, where water is a limiting factor on vegetation growth, the 23% reduction in average rainfall between the southern and northern watersheds should have a noticeable effect. Indeed, rainfall in the 200 to 800 mm yr^-1^ range has previously been found to correlate strongly with woody vegetation cover in this region [1]. However, while this was partially true for the cover of medium and tall tree vegetation, our results suggest that the geologic controls of soil over the availability of this rainfall may be far more important for cover shorter vegetation. Nevertheless the partial control of rainfall over large woody vegetation is important to many aspects of the savanna ecosystem [24]. Consequently, many other ecosystem functions follow the same North-South gradient in rainfall within the KNP. Among these are soil mineralogy and texture [41], herbivore biomass [42,43], and fire behavior [44].

At the watershed level, we found minimal observable spatial correlations between the cover of medium- (on basalt) and large-sized trees within the watersheds. This is in agreement with predictions from competition-based models of tree interactions, which can lead to more regularly-spaced individuals [45]. A sufficiently large spacing between individuals would map to the horizontal patterns we observed in the variograms (Fig 4). Yet, from observations in this and similar systems, we know that larger tree vegetation can cluster together around water-rich areas and/or where water holding capacity is increased [46,47]. This apparent contradiction is reconciled with the understanding that such clusters are relatively rare and the breadth of an individual cluster is typically smaller in scale than the spatial resolution of our variograms (computed at 75 m distance lag intervals).

Conversely, for the shorter vegetation classes, there was considerable spatial correlation among their cover values within 500 m. While climate and substrate explained much more of the variation in the cover of these smaller height classes (Table 1), there was little difference in variogram shape between the four watersheds (Fig 4). Similarly, the variograms for density of detected crown tops takes nearly the same shape. This is an interesting finding, since the four watersheds are clearly different in nearly every measured environmental characteristic (Fig 3). While we know the fire regimes are different between the watersheds (Fig 3), the behavior of herbivores may drive the formation of such patterns. Due to their size, herding behavior, and dietary need to move constantly [48], elephants in particular cause drastic vegetation change [49] and could impart such spatial structure. Another explanatory mechanism could be the existence of a plant-plant facilitation effect that functions in a consistent manner across the landscape. There is evidence that woody plants can have positive interactions with one another and with grasses [15,50]. However, for this mechanism to cause the consistent spatial patterns found here, it would have to be indifferent to the changes in climate, substrate, terrain and species composition represented in our study area.

Vegetation height was the only metric for which spatially-aligned trends were tied with substrate and climate. Because height is controlled in a large part by water availability and transport limitations [51], topo-edaphic factors that affect water availability should exert the most control over vegetation height. In the variograms of vegetation height (Fig 4), it is the granite watersheds that have the shortest range. This observed spatial pattern aligned with our understanding of catena morphology in granite watersheds because catena lengths (stream to crest) are much shorter in this substrate. Major changes in soil texture occur at the toeslope [52], which lead to large differences in vegetation [13]. Additionally, the effects of catenas on woody canopy structure increase in magnitude in wetter climates [52,53]. We also observed that relative slope position is an influential variable in the models of vegetation height in the granite watersheds (S2 Fig)

Using several environmental variables, the models of vegetation structure differed greatly in their ability to explain the variation within each of the four watersheds. In the highly variable environment within the watersheds, the models explained a reasonable share of the variation in woody structure. However, the incorporation of an autocovariate term into the models nearly doubled the R^2^ values for the models of shrub and small tree cover and stem density. The incorporation of spatial trend variables has similarly improved other finer-scale ecological models [54,55]. However, the observed variation and autocorrelation is greatly reduced at scales larger than 1 km [56]. Given that model performance was weak even after the addition of an all-encompassing ACV term, it is very likely that there is simply a large amount of variation inherent in these structural metrics at the fine resolution of our data. One cause could be small errors in the LiDAR-derived ground elevation map which influence results concerning shorter vegetation classes. Moreover, the binary nature of taller tree presence at fine scales, combined with the low overall cover of such vegetation in these watersheds, naturally leads to increased variation. However, any improvement from the ACV terms implies that a significant amount of spatial patterning remained in several models after all environmental variables were included. This suggests that one or more important environmental variables were missing from our analyses.

Previous research provides hints about the variables that may be adding spatial patterning to woody vegetation structure not determined by our analysis. First, rainfall can vary over scales as small as 100 m or less [57], and if any areas are more heavily exposed to rainfall over a couple of years, such hotspots could lead to regions of taller or denser vegetation. Second, soil type determines both the portion of rainfall that is available to plants and general nutrient availability. In turn, these properties exert strong control over plant growth [58,59]. The importance of soil was confirmed in the wet granite watershed models, where soil texture information was available and proved most influential. Third, while we could include fire frequency as a factor, the behavior of an individual fire can also increase the spatial similarity of vegetation structure at smaller scales within a landscape. Fire intensity is a strong determinant of whether taller woody vegetation is killed [60], and higher intensity fires can result in significant drops in woody tree cover [17,61,62]. Fourth, herbivores compete with fire in their overall impact on woody vegetation [20,21]. Elephants, the most influential of these herbivores in the four watersheds, have been identified as responsible for much woody cover change in KNP [49,63]. Also, the effects of elephants and fire are interactive [28], potentially adding more spatially discrete spatial patterning to vegetation structure. A fifth important missing factor is vegetation species composition. The mix of woody and herbaceous species will determine how these two growthforms compete with one another to shape vegetation structure [8,16]. In addition, different species respond differently to similar conditions [64]. The mixture can also affect the vegetative response to outside influences such as fire and herbivore activity [65–67]. In a feedback loop, the composition of plant species affects the behavior of fire and herbivores [68,69]. These five factors, in addition to other factors, may mediate vegetation structure at various spatial scales, showing up in our models as spatially correlated errors.

## Conclusion

We combined LiDAR-derived maps of vegetation and topography with local information of four entire African savanna watersheds to examine how factors influencing vegetation structure work at scales ranging from fine (< 50 m) to geologic (> 100 km). Substrate was clearly more influential than climate, yet fewer clear patterns emerged beyond theseregional controls. While models incorporating topography, hydrology and fire history explained a significant portion of the structural in some watersheds, unknown factors lumped into a single autocovariate term with a range of 50–450 m were often as or more important. This suggests that the strength and range of influential environmental factors differ from site to site. Further study is needed to better understand how multiple environmental factors combine at different scales to determine vegetation structure and how vegetation may respond to future changes in environment. We believe that this could be best accomplished using a combination of spatially-explicit remote sensing and highly-specific, controlled field based approaches.

## Supporting Information

S1 Fig Residual correlation before and after autocovariate term inclusion in models. Spline-interpolated correlograms of the residuals of selected all six full models of the wetter granite watershed, both before (red) and after (blue) the inclusion of an autocovariate (ACV) term into the model. Dashed lines show the maximum and minimum values across six resamples of the data. A rug plot is added with all point pair distances to show the distances between paired sample locations. The ACV excelled at removing any remaining spatial autocorrelation from the models. Before adding the ACV term, most remaining spatial correlation was limited to the first 100 to 200 m. Proportion vegetation is defined as the proportion of area in a 16.8 x 16.8 m window of the vegetation height map that is in the given height class: 0.5 to 2.5 m (shrub), 2.5 to 5.0 m (small tree), 5.0 to 10.0 m (medium tree), and >10.0 m (large tree).
(TIF)

S2 Fig Importance of individual environmental factors to the models. Factor importance, quantified using the difference in R^2^ values between the full model and the model without the indicated variable (and its associated interaction terms), for each watershed and response variable. Listed variables are: slope (SLP), aspect (ASP), relative slope position (RSP), distance to order 1,2 and 3 stream (DO1, DO2, and DO3), fire return interval (FRI), fire wait time (FWT), topographic wetness (WET), and soil type (STP, available only on the southern granite site). Proportion vegetation is defined as the proportion of area in a 16.8 x 16.8 m window of the vegetation height map that is in the given height class: 0.5 to 2.5 m (shrub), 2.5 to 5.0 m (small tree), 5.0 to 10.0 m (medium tree), and >10.0 m (large tree).
(TIF)

S1 File Sample point data used in the analyses described in the manuscript. (ZIP)

S1 Table Environmental traits of the four focal watersheds. (DOCX)
