# Supplementary figures and images for: Multiple Scales of Control on the Structure and Spatial Distribution of Woody Vegetation in African Savanna Watersheds
Source: PLoS One. 2015 Dec 14;10(12):e0145192. doi: 10.1371/journal.pone.0145192 (PMC4679339; doi:10.1371/journal.pone.0145192)

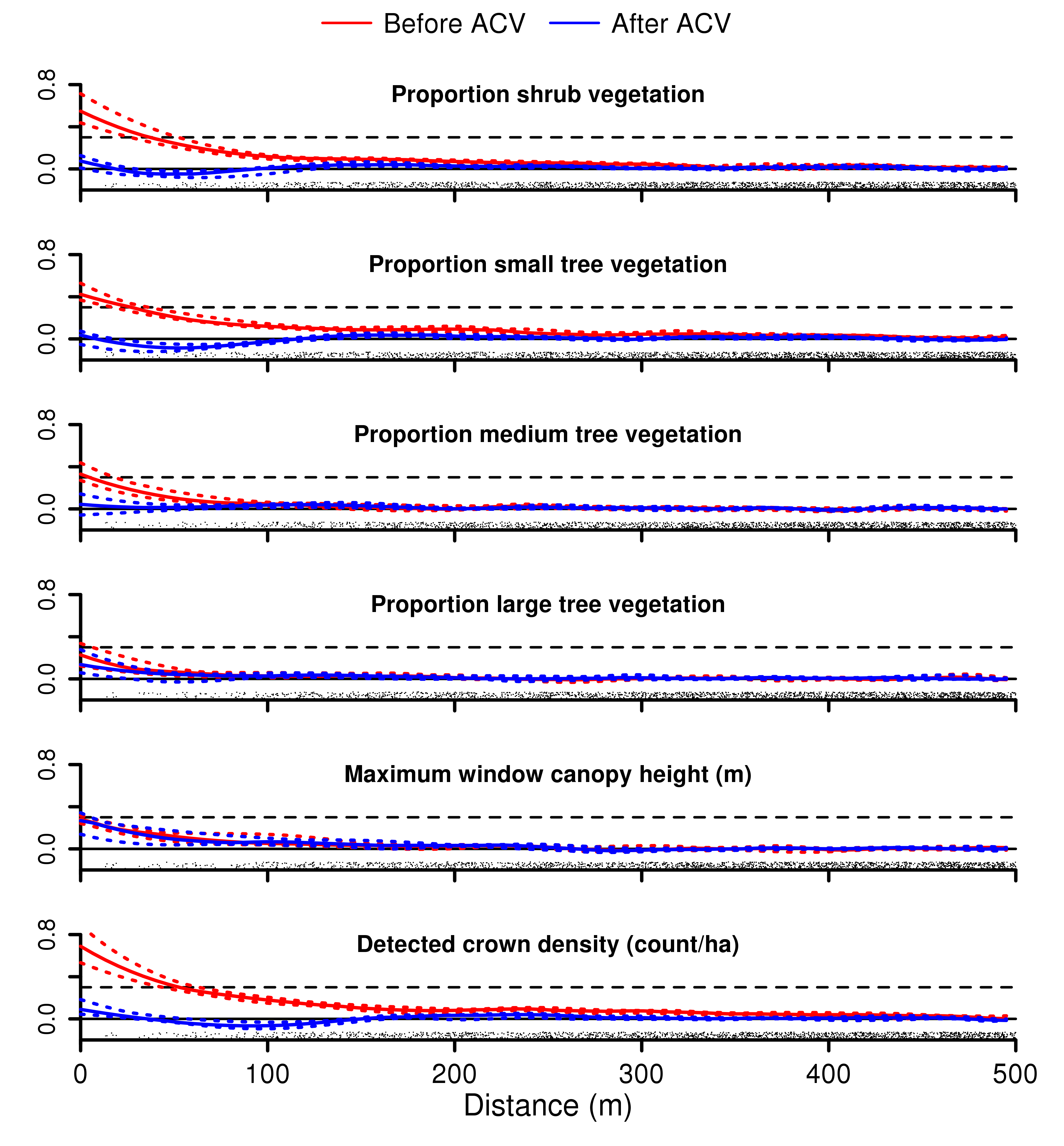

Supplement: S1 Fig — Spline-interpolated correlograms of the residuals of selected all six full models of the wetter granite watershed, both before (red) and after (blue) the inclusion of an autocovariate (ACV) term into the model. Dashed lines show the maximum and minimum values across six resamples of the data. A rug plot is added with all point pair distances to show the distances between paired sample locations. The ACV excelled at removing any remaining spatial autocorrelation from the models. Before adding the ACV term, most remaining spatial correlation was limited to the first 100 to 200 m. Proportion vegetation is defined as the proportion of area in a 16.8 x 16.8 m window of the vegetation height map that is in the given height class: 0.5 to 2.5 m (shrub), 2.5 to 5.0 m (small tree), 5.0 to 10.0 m (medium tree), and >10.0 m (large tree). (TIF) [file pone.0145192.s001.tif]

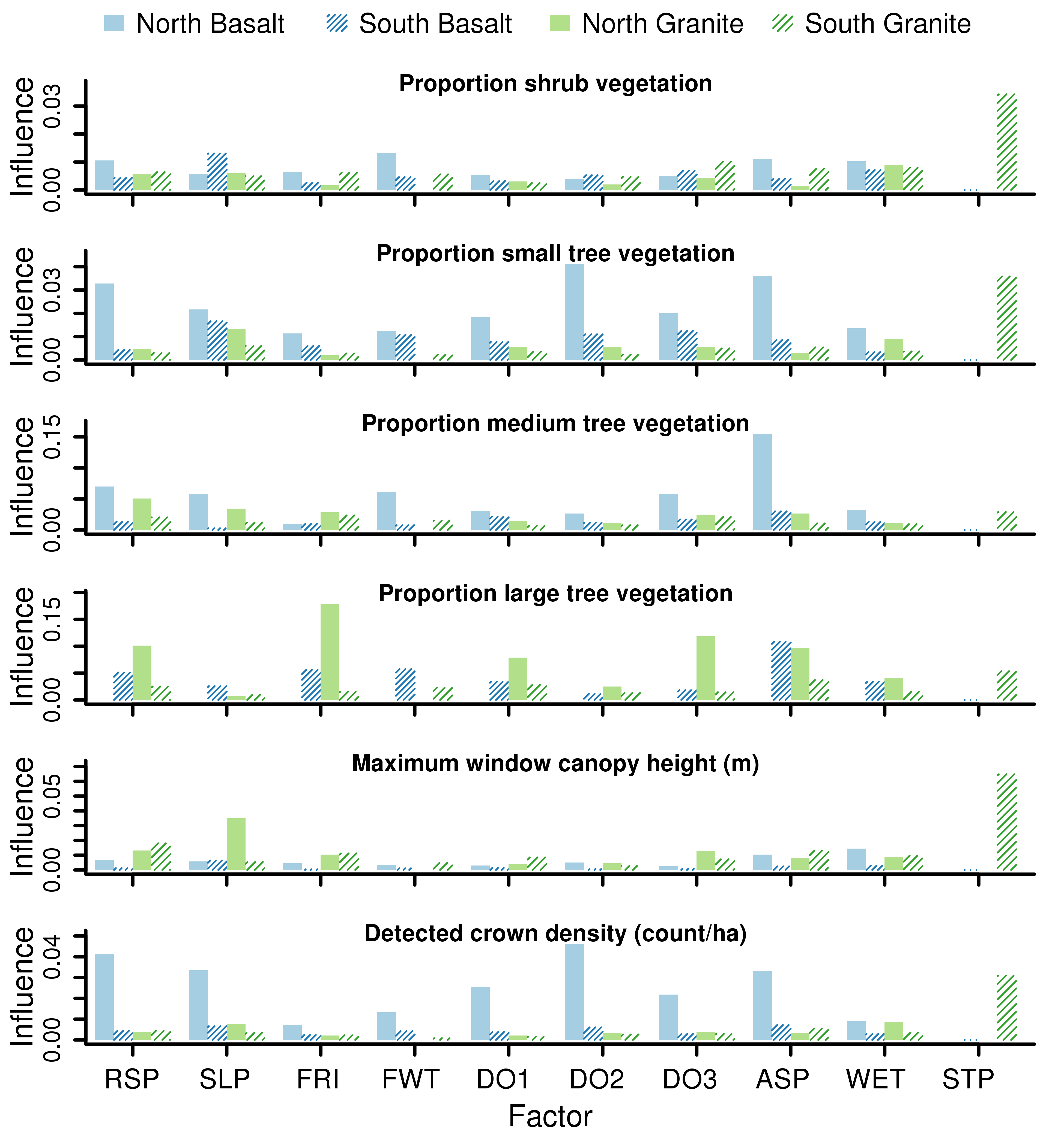

Supplement: S2 Fig — Factor importance, quantified using the difference in R2 values between the full model and the model without the indicated variable (and its associated interaction terms), for each watershed and response variable. Listed variables are: slope (SLP), aspect (ASP), relative slope position (RSP), distance to order 1,2 and 3 stream (DO1, DO2, and DO3), fire return interval (FRI), fire wait time (FWT), topographic wetness (WET), and soil type (STP, available only on the southern granite site). Proportion vegetation is defined as the proportion of area in a 16.8 x 16.8 m window of the vegetation height map that is in the given height class: 0.5 to 2.5 m (shrub), 2.5 to 5.0 m (small tree), 5.0 to 10.0 m (medium tree), and >10.0 m (large tree). (TIF) [file pone.0145192.s002.tif]
