# Supplementary material for: Multiple Scales of Control on the Structure and Spatial Distribution of Woody Vegetation in African Savanna Watersheds
Source: PLoS One. 2015 Dec 14;10(12):e0145192. doi: 10.1371/journal.pone.0145192 (PMC4679339; doi:10.1371/journal.pone.0145192)
Supplement: S1 Table — (DOCX) [file pone.0145192.s004.docx]

**S1 Table.** **Environmental traits of the four focal watersheds.**

| Watershed | Substrate | Region | Area | Rainfall | Latitude range | Longitude range |
| --- | --- | --- | --- | --- | --- | --- |
|  |  |  | --ha-- | --mm/yr-- | --deg.-- | --deg.-- |
| Mooiplaas | Basalt | North | 10826 | 447 (244,730) | -23.542, -23.402 | 31.367, 31.489 |
| Nhlowa | Basalt | South | 4465 | 527 (368,717) | -25.265, -25.193 | 31.891, 31.972 |
| Ngwenyeni | Granite | North | 1987 | 403 (222,712) | -23.863, -23.818 | 31.202, 31.296 |
| Stevenson-Hamilton | Granite | South | 4000 | 566 (307,863) | -25.127, -25.046 | 31.543, 31.606 |
